# Case Report: Prolonged Effects of Short-Term Transcranial Magnetic Stimulation on EEG Biomarkers, Spectral Power, and Seizure Frequency

**DOI:** 10.3389/fnins.2022.866212

**Published:** 2022-06-10

**Authors:** Keith Starnes, Jeffrey W. Britton, David B. Burkholder, Iffat A. Suchita, Nicholas M. Gregg, Bryan T. Klassen, Brian Nils Lundstrom

**Affiliations:** Department of Neurology, Mayo Clinic, Rochester, MN, United States

**Keywords:** TMS, epilepsy, EEG biomarkers, spectral power, EEG, non-invasive brain stimulation

## Abstract

Transcranial magnetic stimulation (TMS) is a non-invasive modality of focal brain stimulation in which a fluctuating magnetic field induces electrical currents within the cortex. It remains unclear to what extent TMS alters EEG biomarkers and how EEG biomarkers may guide treatment of focal epilepsy. We present a case of a 48-year-old man with focal epilepsy, refractory to multiple medication trials, who experienced a dramatic reduction in seizures after targeting the area of seizure onset within the left parietal-occipital region with low-frequency repetitive TMS (rTMS). Prior to treatment, he experienced focal seizures that impacted cognition including apraxia at least 50–60 times daily. MRI of the brain showed a large focal cortical dysplasia with contrast enhancement involving the left occipital-parietal junction. Stimulation for 5 consecutive days was well-tolerated and associated with a day-by-day reduction in seizure frequency. In addition, he was monitored with continuous video EEG, which showed continued and progressive changes in spectral power (decreased broadband power and increased infraslow delta activity) and a gradual reduction in seizure frequency and duration. One month after initial treatment, 2-day ambulatory EEG demonstrated seizure-freedom and MRI showed resolution of focal contrast enhancement. He continues to receive 2–3 days of rTMS every 2–4 months. He was seizure-free for 6 months, and at last follow-up of 17 months was experiencing auras approximately every 2 weeks without progression to disabling seizures. This case demonstrates that rTMS can be a well-tolerated and effective means of controlling medication-refractory seizures, and that EEG biomarkers change gradually in a fashion in association with seizure frequency. TMS influences cortical excitability, is a promising non-invasive means of treating focal epilepsy, and has measurable electrophysiologic effects.

## Introduction

Transcranial magnetic stimulation (TMS) is a non-invasive modality of focal brain stimulation in which a fluctuating magnetic field induces an electrical current within the cortex (Tsuboyama et al., 2020). Each pulse stimulates a small area of tissue and can be used to probe brain states as well to influence cortical excitability. TMS has been applied for depression and presurgical motor and language mapping (Tsuboyama et al., 2020), and is being investigated for its potential as a therapeutic tool in epilepsy (Theodore, 2003; Joo, 2012; Sun et al., 2012; Cooper et al., 2018; Starnes et al., 2019). We report a case of a patient with focal epilepsy, refractory to multiple medication trials and presenting with significant seizure burden, who has experienced a remarkable period of seizure freedom after focal, MRI-guided TMS therapy.

## Case Description

**Figure 1:**
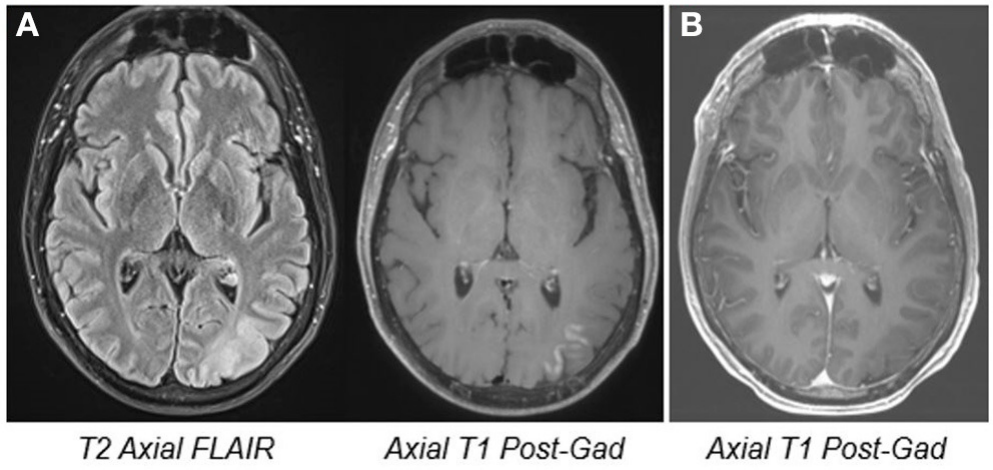
MRI images. Pre- **(A)** and 6 month post-treatment MRI **(B)** showing left occipital cortical thickening and blurring of gray-white junction, implying the presence of a focal cortical dysplasia. The pre-treatment cortical enhancement resolved at the follow-up study.

A 48-year-old man with focal epilepsy, intractable since onset at age 12, presented to our institution for evaluation. His seizures were treated with a combination of valproic acid and carbamazepine. His typical seizure frequency was once or twice monthly. However, starting 2 months prior to presentation, he had experienced a persistent exacerbation of his seizure frequency to 50–60 times daily. Seizure duration was typically 20–30 s, with semiology of feeling unwell and anxious, an illusory auditory sensation, diplopia and oscillopsia, apraxia, and inability to follow commands. Brain MRI showed a focal cortical dysplasia in the left occipital lobe with surrounding cortical enhancement (Figure 1A). CSF evaluation was negative for inflammatory markers or the presence of neural antibodies.

He was admitted to the Epilepsy Monitoring Unit (EMU), where focal seizures were recorded occurring 7–8 times per hour (nearly 200 seizures per day), arising maximally from the left occipital head region near electrode O1 (**Figure 3A**). During the seizures, the patient could respond and perform some calculations, and had no visual field impairment but could not obey simple motor commands or mimicking hand movements.

**Figure 2:**
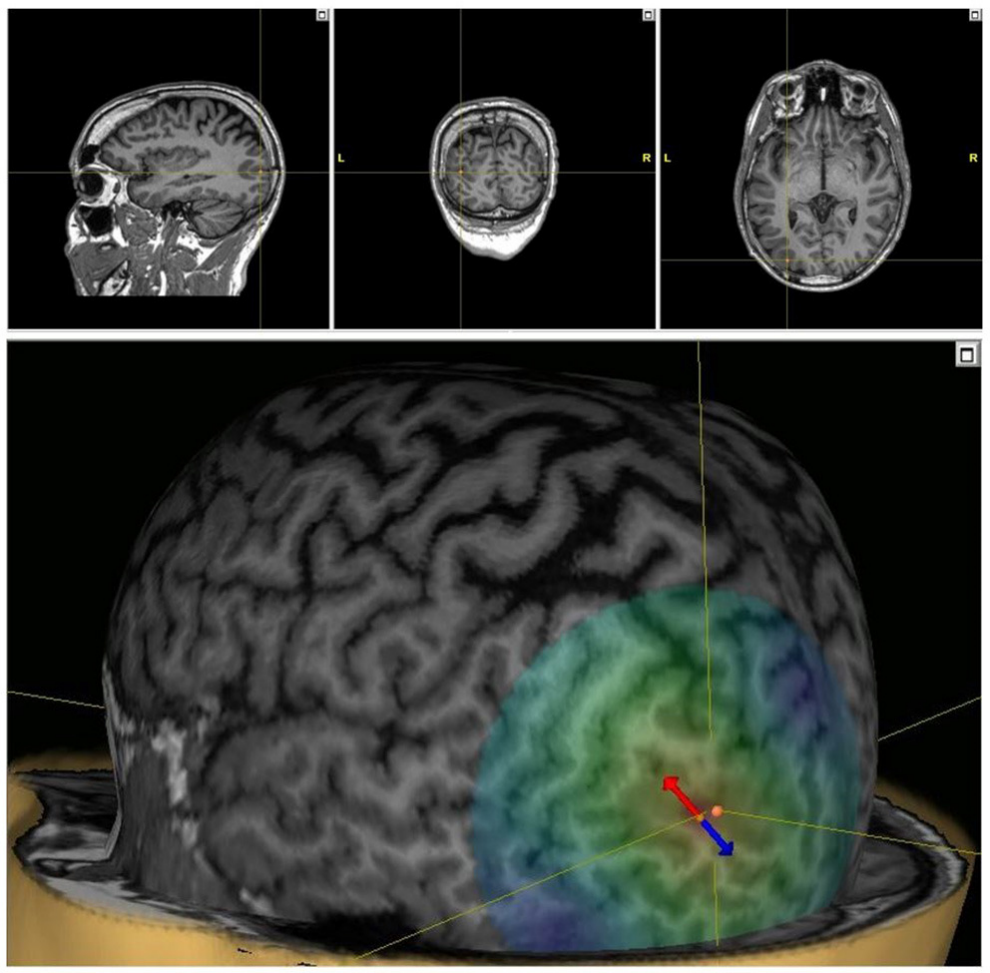
TMS targeting. Stereotactic TMS targeting using the patient's MRI in 3 planes (top) and 3D model (bottom). The dipole of the TMS pulses is indicated, with the arrows indicating the direction of the induced electric field (red cathodal, blue anodal).

**Figure 3:**
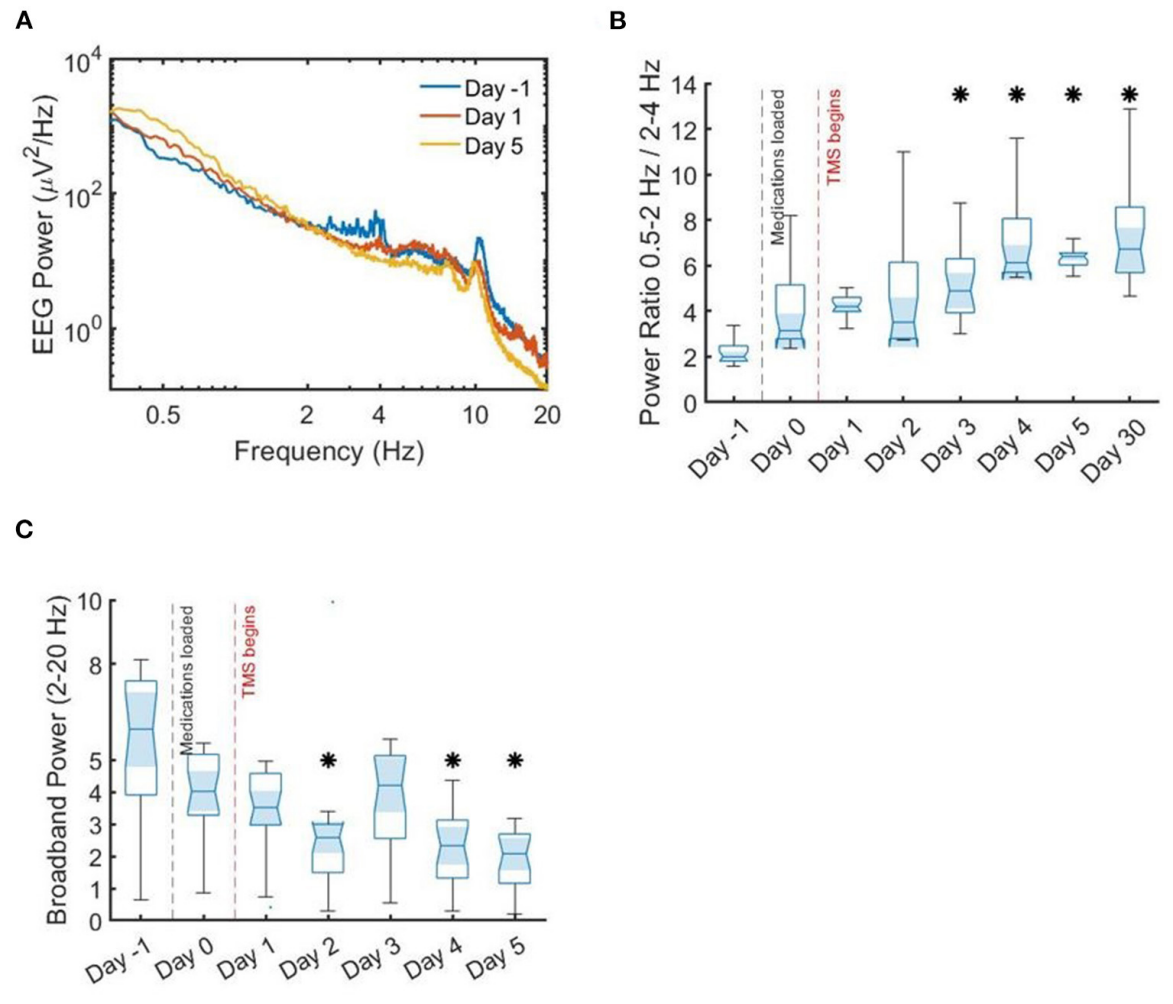
Spectral power density and ratios. Median spectral power density and power ratios. **(A)** Median spectral power density across all channels. There is an inflection point around 2 Hz, with lower frequencies showing more power after stimulation initiation. **(B)** Ratio of slow delta activity to faster delta activity, showing a significant increase in median power by the end of stimulation as compared to prior. **(C)** Broadband power, again showing a significant difference in median by the end of stimulation therapy as compared to before. Asterisks indicate statistical significance by Wilcoxon rank-sum test as compared to prior to stimulation (day 0).

Medication loads with levetiracetam and lacosamide did not improve seizures over the following 24 h, and lacosamide was discontinued. Beginning on day three following admission (“Day 1” of treatment), he was treated with MRI-guided 1 Hz repetitive TMS (Nexstim NBS 5) targeting the left occipital region over 5 consecutive days while undergoing continuous EEG monitoring (Figure 2). MRI-based stereotaxis assisted in precise targeting and stimulus delivery over the 5-day period. Each day starting at ~1 pm in the afternoon, 1,800 pulses of 1 Hz stimulation were provided over 30 mins. Stimulation intensity was determined as a percentage of resting motor threshold (rMT). Initially, stimulation was started at 100% of rMT. However, during the first 10 mins of stimulation, the patient complained of 5/10 pain. Intensity was lowered to 90% of rMT for each subsequent session, and stimulation was well-tolerated without complaint thereafter. There was a gradual improvement in seizure frequency and duration over his 8-day hospitalization (Figure 3). By day 3 of TMS treatment, seizure frequency was 0–5 per hour, and by day 5 it was 0–4. He was discharged to home after completion of stimulation.

**Figure 4:**
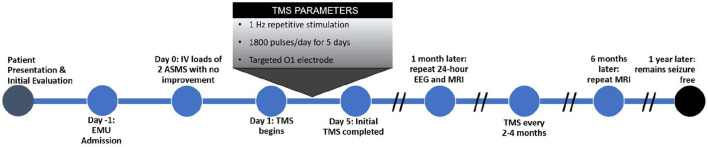
Timeline.

After dismissal from the hospital, the patient was seizure-free for 6 months, and was able to stop valproic acid and levetiracetam, and reduce the dose of carbamazepine. Ambulatory EEG on day 31 (post-stimulation) showed no seizure activity over 24 h. One-month follow-up MRI showed resolution of left occipital cortical enhancement. At latest follow up of 17 months, he was experiencing auras approximately every 2 weeks without progression to disabling seizures. He has returned five times for additional rTMS treatments, for a mean follow-up interval of every 3.4 months (Figure 4). Subsequent treatments are provided as 2–3 days of 1 Hz rTMS with the same parameters as the initial therapy.

## Diagnostic Assessment

In addition to raw EEG review, EEG digital analysis was performed. Seizures were automatically detected and quantified for the entire recording using Persyst 14 (Persyst Development Corporation, San Diego, CA) (Scheuer et al., 2021). Automated seizure detections decreased over the course of therapy (Figure 3). Specifically, in the 24 h following the intravenous infusion of antiseizure medications, his seizure burden increased by ~20%. Following the initiation of his 5-day rTMS treatment course, his seizure burden decreased by 5–30% per day.

**Figure 5:**
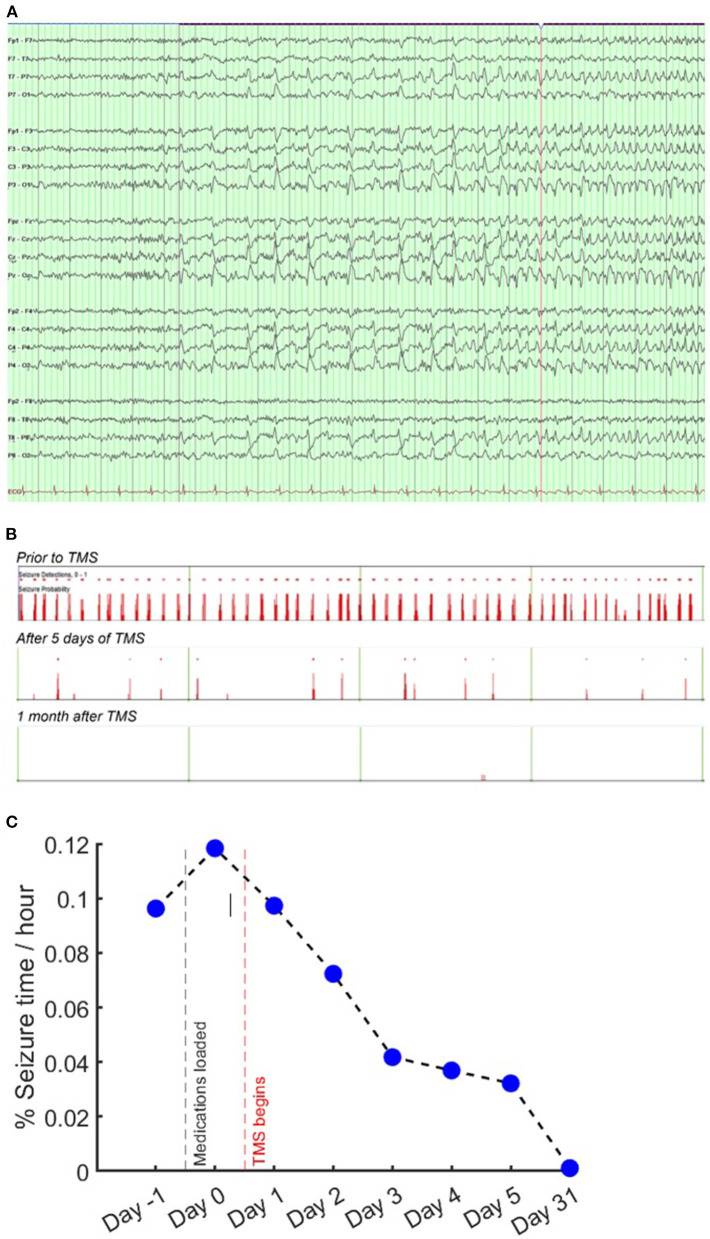
EEG and seizure frequency. EEG seizure onset and automated seizure detections. **(A)** EEG seizure onset on longitudinal bipolar montage. EEG settings: low frequency filter 7 Hz, high frequency filter 70 Hz, sensitivity 7 uV/mm. **(B)** 16-h segments showing automated seizure detections (red bars) prior to stimulation; after 5 days of stimulation; and 1 month after TMS. **(C)** Percentage of time spent in seizure, showing an initial 22% increase after medication load, followed by day-by-day reduction after TMS treatment began.

For the analysis of power spectra, 256-Hz sampled EEG segments using all 24 channels of an extended 10–20 EEG montage taken from the first 2 h of sleep each night were used. The segments were comprised of N1–N2 sleep. The data were band-pass filtered between 0.5 and 55 Hz using a fourth-order Butterworth filter; 55 Hz was chosen as the low-pass filter to avoid 60 Hz artifact. Spectral density was estimated using Welch's method. Median power per frequency bin across all channels was plotted for the day prior to TMS therapy, the day stimulation began, and on the last day of the initial TMS therapy (Figure 5A).

Based upon prior work showing that low frequency interictal activity is a useful biomarker for localizing seizure onset zone (SOZ) and predicting outcome of surgical intervention (Lundstrom et al., 2019a, 2021; Baldini et al., 2020), we analyzed delta activity (0.5–4 Hz) for the sampled EEG data corresponding to each day. Upon review of the day-by-day EEG power, we noticed an inflection point around 2 Hz; on this basis, similar to previous research (Lundstrom et al., 2019a, 2021), we compared the ratio of 0.5–2 Hz infraslow activity to 2–4 Hz delta activity (Figure 5B). The value of this ratio showed gradual decrement over the course of the hospitalization, with a significant difference in median power at the conclusion of treatment as compared to before (*p* < 0.0001 by Wilcoxon rank sum test). Broadband spectral power from 2 to 20 Hz was gradually decreased with stimulation (Figure 3C), also with a significant difference in medians by the fifth day of stimulation (*p* < 0.0001 by Wilcoxon rank sum test). These changes were coincident with improvement in seizure frequency (Figure 3). These results are consistent with previous work showing that low frequency activity (<2 Hz) is decreased near the seizure onset zone, while higher frequency activity (2–50 Hz) is increased (Lundstrom et al., 2019a, 2021). In this case, TMS therapy was associated with an increase in 0.5–2 Hz activity and decrease in 2–50 Hz activity, thereby leading to a power spectral signature similar to non-seizure onset zone cortical brain regions.

## Discussion

Our report describes a case of refractory focal seizures with robust response to repetitive TMS (rTMS). Low-frequency, rTMS has emerged as a potential treatment for epilepsy. Although potentially effective in other situations, evidence has shown that rTMS may be particularly well-suited for cases of superficial, cortically-based focal epilepsies that are amenable to stimulation targeting (Tsuboyama et al., 2020), as in this case. In this patient, EEG and imaging data were used to select a superficial cortical target, which was confirmed using stereotaxis. The MRI finding of cortical enhancement has been described as a peri-ictal phenomenon (Williams et al., 2017), and the resolution at the follow-up study is most likely related to improved seizure frequency.

TMS influences brain states, exciting neurons and triggering action potentials, and inducing effects which approximate long-term potentiation or long-term depression (Huerta and Volpe, 2009). While these effects are exerted focally, TMS may influence circuit-level patterns such as underlying network oscillations, blood flow, as well as gene and protein regulation (Yamamoto et al., 2002; Huerta and Volpe, 2009; Sunderam et al., 2010). TMS also activates more than just brain tissue, inducing action potentials in extracranial tissues, CSF “eddy currents,” and audio-evoked potentials due to the “click” when stimulation is activated (Conde et al., 2019) – potential confounders when interpreting the mechanisms of TMS. TMS-EEG has been used to investigate cortical excitability in varying brain states (Casali et al., 2013) as well as the electrophysiologic effects of antiseizure medications (Darmani et al., 2016).

In this patient, rTMS altered the spectral density of continuous EEG recordings. There was a reduction in broadband frequency power and a relative increase in infraslow delta power, along with a reduction in seizure frequency. Other studies have shown a similar effect of brain stimulation on spectral power (Kinoshita et al., 2005; Lundstrom et al., 2019b; Westin et al., 2019) and spike rate (Kinoshita et al., 2005; Sun et al., 2012; Lundstrom et al., 2018). This change in spectral power has also been seen in correlation with reduced frequency of interictal discharges (Westin et al., 2019), which in turn is associated with decreased seizure frequency in other patients undergoing brain stimulation for epilepsy (Velasco et al., 2000; Lundstrom et al., 2018). The increase in infraslow activity may be an EEG biomarker for the SOZ and surgical prognostication (Lundstrom et al., 2021). There is a growing body of evidence for the importance of very slow EEG activity in brain network dynamics and functional connectivity, and the impact that these fluctuations have on a variety of neurocognitive and neuropsychiatric disease states (Jones et al., 2012; Grooms et al., 2017; Li et al., 2019; Wirsich et al., 2020). Patients with epilepsy have increased slow-wave activity while in the resting state (Boly et al., 2017), and these underlying network oscillations likely exert a strong influence on seizure generation (Moran et al., 2013; Jirsa et al., 2014; Gregg et al., 2020).

There is a need for biomarkers to gauge the effect of brain stimulation and predict clinical response. While TMS may reveal information on brain states and network dynamics, it is remains uncertain whether these changes in electrophysiological measurements are predictive of response to stimulation (Westin et al., 2019). There have been reports of successful treatment of refractory focal status epilepticus with TMS (Liu et al., 2013; Zeiler et al., 2015). Brain stimulation impacts network variability and dynamics, and these influences can be measured using functional imaging modalities (Ji et al., 2017; Liao et al., 2019). This case report suggests that they could also be measured by EEG biomarkers

In this case, continuous EEG recording was available over a substantial period prior to, during, and after brain stimulation, and follow-up imaging and clinical and electrophysiological data confirmed durable improvements in epilepsy severity and EEG power. This report adds to evidence that brain stimulation may exert therapeutic effects at least in part by reducing aberrant network fluctuations and by promoting stability and normal connectivity. These objective measurements may not be available for every patient considering rTMS treatment for epilepsy. Further work is needed to determine if these measures of cortical excitability are applicable across a variety of patient populations, and to identify other potential biomarkers which may be more widely accessible and could predict treatment response.

## Conclusion

In this patient with refractory lesional focal epilepsy, rTMS was well-tolerated and effective in controlling seizures whereas medications were not. TMS influences cortical excitability, is a promising non-invasive means of treating focal epilepsy, and has measurable effects on EEG. Further investigation is needed to determine useful biomarkers for non-invasive brain stimulation. From the patient's perspective, he mentions that although receiving this treatment was initially intimidating, in retrospect it was the best thing that has happened in his life aside from meeting his wife. He feels that he has regained his life back, and he is looking forward to new opportunities.

## Data Availability Statement

The data analyzed in this study will be shared upon request to the corresponding author. Requests to access these datasets should be directed to starnes.donnie@mayo.edu.

## Author Contributions

All authors listed have made a substantial, direct, and intellectual contribution to the work and approved it for publication.

## Funding

This research was supported by NIH NINDS K23NS112339 (BL).

## Conflict of Interest

BL was a named inventor for intellectual property developed at Mayo Clinic, licensed to Cadence Neuroscience Inc., and waived contractual rights to royalties. BL was a principal investigator, and NG was a co-investigator for the Medtronic Deep Brain Stimulation Therapy for Epilepsy Post-Approval Study (EPAS), Neuropace RNS System Responsive Stimulation for Adolescents with Epilepsy (RESPONSE) Study, and Neuroelectrics tDCS for Patients with Epilepsy Study. BL was an investigator for Mayo Clinic Medtronic NIH Public Private Partnership (UH3-NS95495). Mayo Clinic has received consulting fees on behalf of BL from Epiminder, Medtronic, and Philips Neuro. The remaining authors declare that the research was conducted in the absence of any commercial or financial relationships that could be construed as a potential conflict of interest.

## Publisher's Note

All claims expressed in this article are solely those of the authors and do not necessarily represent those of their affiliated organizations, or those of the publisher, the editors and the reviewers. Any product that may be evaluated in this article, or claim that may be made by its manufacturer, is not guaranteed or endorsed by the publisher.
